# Understanding Financial Market States Using an Artificial Double Auction Market

**DOI:** 10.1371/journal.pone.0152608

**Published:** 2016-03-31

**Authors:** Kyubin Yim, Gabjin Oh, Seunghwan Kim

**Affiliations:** 1 Nonlinear and Complex System Laboratory, Department of Physics, Pohang University of Science and Technology, Pohang 790-784, Republic Of Korea; 2 Division of Business Administration, Chosun University, Gwangju 501-759, Republic Of Korea; East China University of Science and Technology, CHINA

## Abstract

The ultimate value of theories describing the fundamental mechanisms behind asset prices in financial systems is reflected in the capacity of such theories to understand these systems. Although the models that explain the various states of financial markets offer substantial evidence from the fields of finance, mathematics, and even physics, previous theories that attempt to address the complexities of financial markets in full have been inadequate. We propose an artificial double auction market as an agent-based model to study the origin of complex states in financial markets by characterizing important parameters with an investment strategy that can cover the dynamics of the financial market. The investment strategies of chartist traders in response to new market information should reduce market stability based on the price fluctuations of risky assets. However, fundamentalist traders strategically submit orders based on fundamental value and, thereby stabilize the market. We construct a continuous double auction market and find that the market is controlled by the proportion of chartists, *P*_*c*_. We show that mimicking the real state of financial markets, which emerges in real financial systems, is given within the range *P*_*c*_ = 0.40 to *P*_*c*_ = 0.85; however, we show that mimicking the efficient market hypothesis state can be generated with values less than *P*_*c*_ = 0.40. In particular, we observe that mimicking a market collapse state is created with values greater than *P*_*c*_ = 0.85, at which point a liquidity shortage occurs, and the phase transition behavior is described at *P*_*c*_ = 0.85.

## Introduction

Because of its crucial role, asset pricing has long been a subject of study in financial markets, and a grasp of investor behavior is important to understand its fundamental mechanisms. No theory in the fields of economics or finance explains all aspects of the price mechanism because financial markets are extremely complex systems and are characterized by various market states, for example, normal and abnormal states. To fully understand the price dynamics of risky assets, we must understand the nature of the diverse states of financial markets. The celebrated scholars Eugene F. Fama and Robert J. Shiller received the 2013 Nobel Prize in Economic Sciences for furthering understanding of the fundamental features of asset pricing in financial markets [1].

### From Efficient Market Hypothesis to Behavioral Finance

Eugene F. Fama proposed the Efficient Market Hypothesis (EMH), which is widely accepted as the foundational economic theory of asset pricing models, including the pricing of both underlying assets and the options related to such assets [2, 3]. In the EMH, “efficient” means information efficiency in the market. The EMH assumes that all investors in the financial markets are rational and that these investors cannot predict future market prices using past financial market information due to perfect information in the market. Furthermore, Fama focuses on the role of the capital markets, where all information about the economy is aggregated, and on the financing of firms using the capital markets as an efficient channel of external financing. With the global development of capital markets, the EMH has been the underlying theory used in corporate finance and financial management as well as asset pricing.

However, Robert J. Shiller proposed “behavioral finance”; according to this theory, irrational traders can predict future market prices using past information about the price movements of risky assets or by examining psychological effects on investors [4]. Furthermore, in behavioral finance, other factors such as herding and framing affect market factors such as prices, returns, and volumes, which are important factors in asset pricing. Behavioral finance has been developed further by a number of other economists and is well established in the mainstream financial economics literature [5–11]. In addition, many market microstructure models have incorporated both the EMH and behavioral finance [12–20]. Moreover, studies at the micro level that utilize high-frequency data from financial markets have also contributed to the asset pricing and market microstructure field [21–25].

Unfortunately, the essential assumption of the EMH is completely different from that of behavioral finance. In other words, most of the research on asset pricing models can explain some of the features of various aspects of the markets by making specific assumptions regarding major factors, such as investor behavior and the dynamics of risky assets. Thus, a firm grasp of the asset pricing mechanism is difficult because asset pricing models comprise a variety of independent theories; a comprehensive understanding of asset pricing is possible only by means of integrated models that map both theories.

### Toward an Integrated Asset Pricing Model: The Agent-Based Model (ABM)

The research using agent-based models (ABM) to understand the stylized facts of asset prices in financial markets has recently shed light on factors that (1) prompt us to question whether the heterogeneity of investors is more important than that of representative agents in this context and that (2) describe how interactions among traders affect the fundamental features of asset prices. As a model of asset prices that considers the heterogeneity of traders, ABM has recently emerged as an alternative approach in asset pricing models. The properties of ABM can be distinguished from other methodologies as follows: (i) ABM provides a linkage from the micro investor level to the macro-market level, and (ii) ABM can generate artificial data that include different market scenarios and heterogeneous agent assumptions. Therefore, an artificial stock market has been created with various scenarios that include not only heterogeneous agent types from zero intelligence models to multi-agent models but also various trading mechanisms from market-clearing systems to order-driven markets [26–44].

To illustrate an alternative asset pricing model that reflects the various market states of real financial markets, we develop an artificial double auction market (ADAM) trading system that is constructed with heterogeneous agents, including fundamentalists and chartists.

### Overview of Artificial Double Auction Market (ADAM)

Traditional asset pricing models in the financial system are typically characterized by one partial aspect among diverse market states, but the dynamics of asset prices in the real financial system have many aspects that can result from the heterogeneity of traders, the interactions among them, and a complicated trading mechanism (the double auction market); thus, we use the ADAM in our study.

In the ADAM, it is assumed that one type of stock is traded. We assume that all agents know the other agents’ type, past price information during their investment time horizon and the current fundamental value $p_{t}^{f}$ of the asset, which we assume follows the geometric Brownian motion defined by

$$\begin{array}{c} \ln\left(p_{t}^{f}\right)-\ln\left(p_{t-1}^{f}\right)=\epsilon_{t},\;\epsilon_{t}∼N\left(0,\sigma_{\epsilon }\right) \end{array}$$ (1)

where $p_{t}^{f}$ denotes the fundamental value at time *t*. The increments of $\ln(p_{t}^{f})$ follow a normal distribution that yields a standard deviation equal to *σ*_*ϵ*_ for the aggregate of the increments over integer time steps.

**Fig 1:**
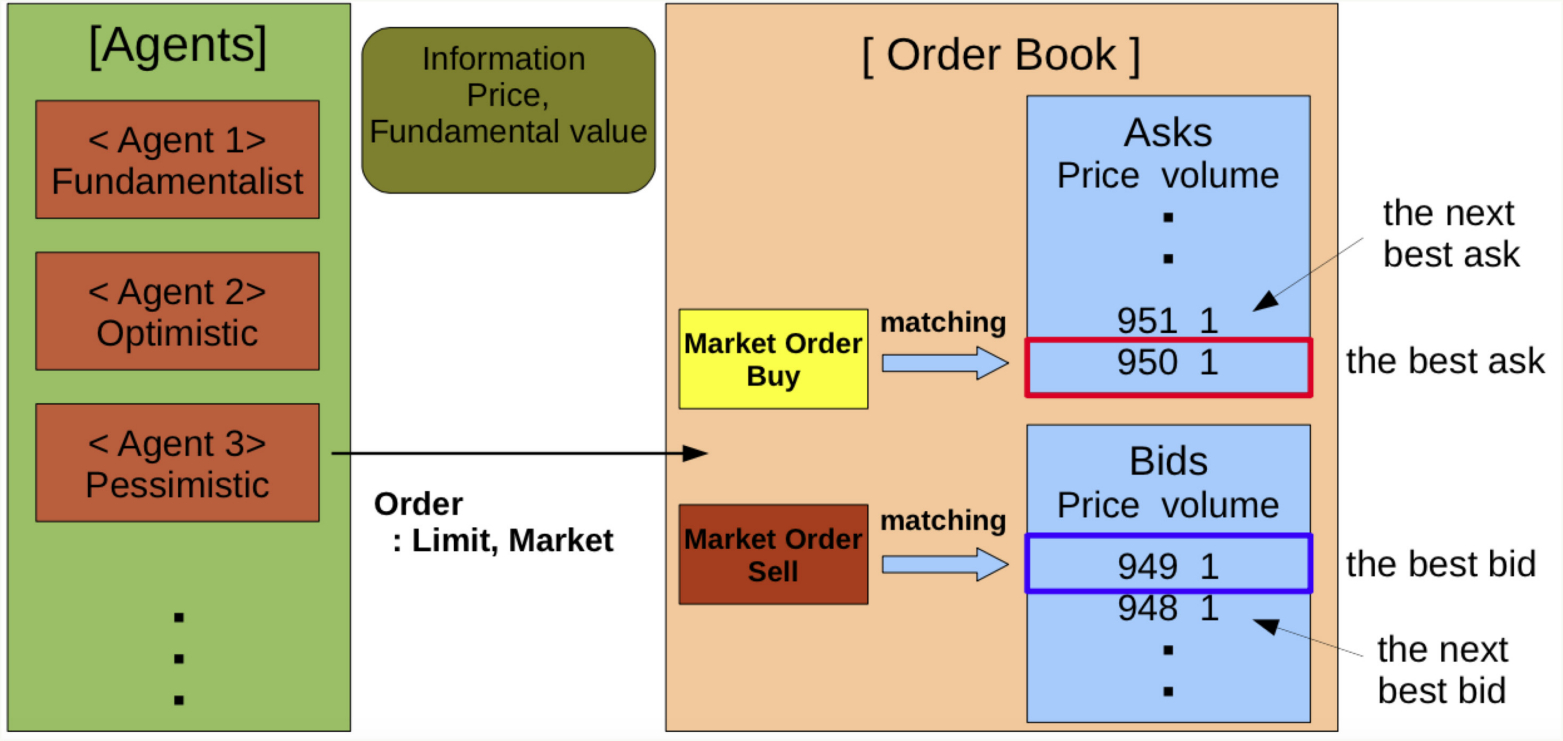
The schema of the trading process incorporated in the ADAM.

Fig 1 describes the schema of the trading process in the ADAM. At the first step of the trading process, an individual agent determines her own type according to switching rules among agent types. In the ADAM, there are two heterogeneous agent types:fundamentalist and chartist. Additionally, chartists are classified into two types according to sentiment, i.e., optimistic or pessimistic. We consider switching rules using transition probabilities, which consist of relative payoff and herding. The details of the agent types are described in the Methods section. After an agent type is determined, one randomly chosen agent forecasts the future price and determines her order. Finally, the agent submits the order, which is determined as either a market order or a limit order under the double auction mechanism. If an agent submits a market order to buy(sell), this market order is matched with a limit order to sell(buy) at the best ask(bid) price, and the market price is determined by the best ask(bid) price. If an agent submits a limit order to buy(sell), this order is stored in a bid(ask) limit order book. This trading process can be divided into two sub-processes: the determining agent type process and the double auction market process. The details of the two sub-processes are also described in the Methods section.

The remainder of this paper is organized as follows. In the Methods section, we describe the details of the ADAM. The Results section describes and discusses the results of the data generated by the model. The Discussion and Conclusions sections summarize the paper and propose avenues for future research.

## Methods

### The Process of Determining the Agent Type

In the ADAM, the trading process begins by determining the agent type. We consider two heterogeneous agent types: the fundamentalist and the chartist. The fundamentalist agent prefers fundamental analysis and corresponds to a fundamental trader in the real financial market [45]. In the ADAM, fundamentalists obtain fundamental value information from fundamental analysis. Generally, the fundamental value of a stock is calculated based on the discounted sum of future profits or the earnings of the company issuing the stock. The movement of this fundamental value is a random walk and is largely unaffected by the trend in the market price. In the ADAM, fundamental value is assumed to follow a Brownian motion, as represented by Eq 1. Therefore, fundamentalism both stabilizes the market and randomizes market prices. Market states driven by fundamentalists will be similar to the efficient markets proposed by Eugene F. Fama. Unlike fundamentalists, chartists are sensitive to the trends or fashions of market prices. Chartists are agents who prefer technical analyses using past price trends and correspond to technical traders in the real financial market [46]. As proposed by Robert J. Shiller, a chartist is an irrational trader and speculator who destabilizes a market. In the ADAM, a chartist is a trend-follower who uses the trends of past prices. Additionally, chartists are classified as one of two types: optimistic or pessimistic. Optimistic(pessimistic) chartists forecast that future prices will be larger(smaller) than the current price.

In most of the artificial stock markets using ABM, fundamentalists and chartists are mixed in their expectations of the spot price, which can be stochastically determined using initial fixed parameters [35, 38, 40]. These models have the advantage of generating the market state by varying the combinations of agent types. However, these models do not provide an adequate description of the dynamic properties of the market microstructure when there is a change in the agent type. To understand the dynamics of the market microstructure when there is a change in the agent type, we segregate the fundamentalists and chartists based on their expectations, and we consider switching rules between them using transition probabilities by modifying the rules of opinion dynamics from previous artificial stock market models [31, 32]. Transition probabilities include herding, which occurs in the interactions between agents, and the profit terms of each agent type. More details regarding the switching rules are presented in Supporting Information (S1 File).

### Double Auction Market Process

After all agents determine their own type, one agent is randomly chosen to participate in trading at any time *t*. The chosen agent *i* forms an expectation about the spot price or the future price ${\hat{p}}_{t,t+\tau^{i}}^{i}$ that will prevail in the time interval (*t*, *t* + *τ*^*i*^), where *τ*^*i*^ denotes the investment time horizon of agent *i*. The future prices of fundamentalist, optimistic and pessimistic agents are as follows:

$$\begin{array}{c} {\hat{p}}_{t,t+\tau^{i}}^{i}\left(fundamentalist\right)=p_{t}^{f}\left(1+N\left(0,\frac{\sigma_{\epsilon }}{\gamma_{f}}\right)\right) \end{array}$$ (2)

$$\begin{array}{c} {\hat{p}}_{t,t+\tau^{i}}^{i}\left(optimistic\right)=p_{t}+\left|N\left(0,\frac{\sigma_{\tau^{i}}}{\gamma_{c}}\right)\right|\sqrt{T^{i}} \end{array}$$ (3)

$$\begin{array}{c} {\hat{p}}_{t,t+\tau^{i}}^{i}\left(pessimistic\right)=p_{t}-\left|N\left(0,\frac{\sigma_{\tau^{i}}}{\gamma_{c}}\right)\right|\sqrt{T^{i}} \end{array}$$ (4)

$$\begin{array}{c} \sigma_{\tau^{i}}=\sqrt{\frac{\sum_{k=1}^{T^{i}}{\left(p_{t-k}-\bar{p}\right)}^{2}}{T^{i}}} \end{array}$$ (5)

$$\begin{array}{c} \bar{p}=\frac{\sum_{k=1}^{\tau^{i}}p_{t-1-k}}{T^{i}} \end{array}$$ (6)

$$\begin{array}{c} T^{i}=\frac{\tau^{i}}{\Delta t} \end{array}$$ (7)

where $p_{t}^{f}$ denotes the fundamental value at time *t*, *γ*_*f*_, *γ*_*c*_ denotes the risk aversion coefficient of fundamentalists and chartists, Δ*t* denotes one simulation time step, and *τ*^*i*^ denotes the investment time horizon of agent *i*. We assume that *γ*_*f*_ is larger than *γ*_*c*_. Additionally, we assume that the *τ*^*i*^ of fundamentalists is larger than that of chartists. These assumptions reflect the characteristic that chartists are more speculative than fundamentalists. $N(0,\frac{\sigma_{\tau^{i}}}{\gamma_{c}})$ is a normal distribution with a mean of zero and $\frac{\sigma_{\tau^{i}}}{\gamma_{c}}$ as the standard deviation. *σ*_*τ*^*i*^_ denotes the standard deviation of prices during [*t*−*τ*^*i*^, *t*). The increment of the simulation time is Δ*t*. Chartists take the standard deviation of future prices from the market data. Optimistic(pessimistic) agents drive the market price to an up-trend(down-trend) based on the standard deviation of the market price. Fundamentalists take the standard deviation of the future price with *σ*_*ϵ*_, which is the standard deviation of the increments of fundamental values.

If the agent expects that the future price will be larger (smaller) than the current price, she decides to buy(sell) one unit of the stock. However, if the agent expects the future price to be the same as the current price, the agent does not submit an order. We assume that the agent is willing to buy (sell) at a price $b_{t}^{i}\left(a_{t}^{i}\right)$ that is lower(higher) than his expected future price ${\hat{p}}_{t,t+\tau^{i}}^{i}$. $b_{t}^{i}$ and $a_{t}^{i}$ are as follows:

$$\begin{array}{c} b_{t}^{i}={\hat{p}}_{t,t+\tau^{i}}^{i}\left(1-k^{i}\right) \end{array}$$ (8)

$$\begin{array}{c} a_{t}^{i}={\hat{p}}_{t,t+\tau^{i}}^{i}\left(1+k^{i}\right) \end{array}$$ (9)

$$\begin{array}{c} Prob\left(k^{i}\right)=\exp\left(-k^{i}/\sigma \right)/\sigma \end{array}$$ (10)

$$\begin{array}{c} E\left[k^{i}\right]=\sigma \;\;,Var\left[k^{i}\right]=\sigma^{2} \end{array}$$ (11)

where *k*^*i*^ is distributed based on an exponential distribution. According to the double auction mechanism, the agent selects a limit order or a market order. If $b_{t}^{i}$($a_{t}^{i}$) is smaller (larger) than the best ask $a_{t}^{q}$(the best bid $b_{t}^{q}$), the agent submits a limit order at the price level $b_{t}^{i}$($a_{t}^{i}$). The best ask $a_{t}^{q}$(the best bid $b_{t}^{q}$) means that the lowest ask(the highest bid) is listed in a limit order book at time t. A limit order is an order to buy or sell a stock at a specific price or better and is stored in an order book that waits for market orders. However, if $b_{t}^{i}$($a_{t}^{i}$) is larger (smaller) than or equal to $a_{t}^{q}$($b_{t}^{q}$), the agent submits a market order at $a_{t}^{q}$($b_{t}^{q}$). A market order is an order to buy or sell a stock at the best available price in a limit order book. When a market order is submitted in the market, a transaction occurs. Until *t* + *τ*^*i*^, limit orders that are unmatched with a market order are removed from the order book.

At any time *t*, the price *p*_*t*_ is given by the price at which a transaction, if any, occurs. If no new transaction occurs, a proxy for the price is given by the average of $a_{t}^{q}$ and $b_{t}^{q}$ so that $p_{t}=\left(a_{t}^{q}+b_{t}^{q}\right)/2$, which is a value that we call the mid-point. If no bid or ask price is listed in the order book, a proxy for the price is given by the previously traded or quoted price. All prices of orders must be positive, and investors can submit limit orders at any price on a prespecified grid, as defined by the tick size Δ.

All agents trade under a budget constraint, and short sales are forbidden. To prevent an overly large change in the market price, the submission of an order that exceeds or is less than 15% of the closed price at a previous time is forbidden. We assume that the volume of submitted orders is always one unit, and one simulation step, denoted by Δ*t*, is 0.01 of time. We assume one trading period is 100 simulation steps (= 100Δ*t* = 1 time).

After trading is completed, all limit orders and market microstructure trajectories are written in artificial data. By analyzing these artificial data, we can trace all of the processes of price formation at a micro-level.

## Results

We employ the ADAM model developed above to understand the asset pricing model on a market microstructure level. According to the numerous results from previous works, the asset price dynamics in a microstructure scope are characterized by several quantities, including the return, volatility, bid-ask spread, and first gap data sets [47–58]. Here, we simulate the artificial data sets using the ADAM model, which can generate significant information for understanding asset price mechanisms, particularly in terms of the heterogeneity of traders. We now proceed with the simulation and analyze the data model generated. A total of 100 simulations are performed with a different random seed. Each simulation is performed with 500 agents in 1,000,000 simulation steps(= 10,000 times). The values of the parameters used for the simulations are as follows:

*σ*_*ϵ*_ = 0.005, *σ* = 0.1, Δ = 0.0005, *p*(*t* = 0) = 300, *p*_*f*_(*t* = 0) = 300, *γ*_*f*_ = 1.0, *γ*_*c*_ = 0.1, and *τ*^*i*^ = 3 [time] for fundamentalists, and *τ*^*i*^ = 1 [time] for chartists. To protect against market distortion in the ADAM model, we construct a market collapse state from which to recover to other states by virtue of the rule that forbids trading in excess or below 15% of the market price in previous trading periods.

**Fig 2:**
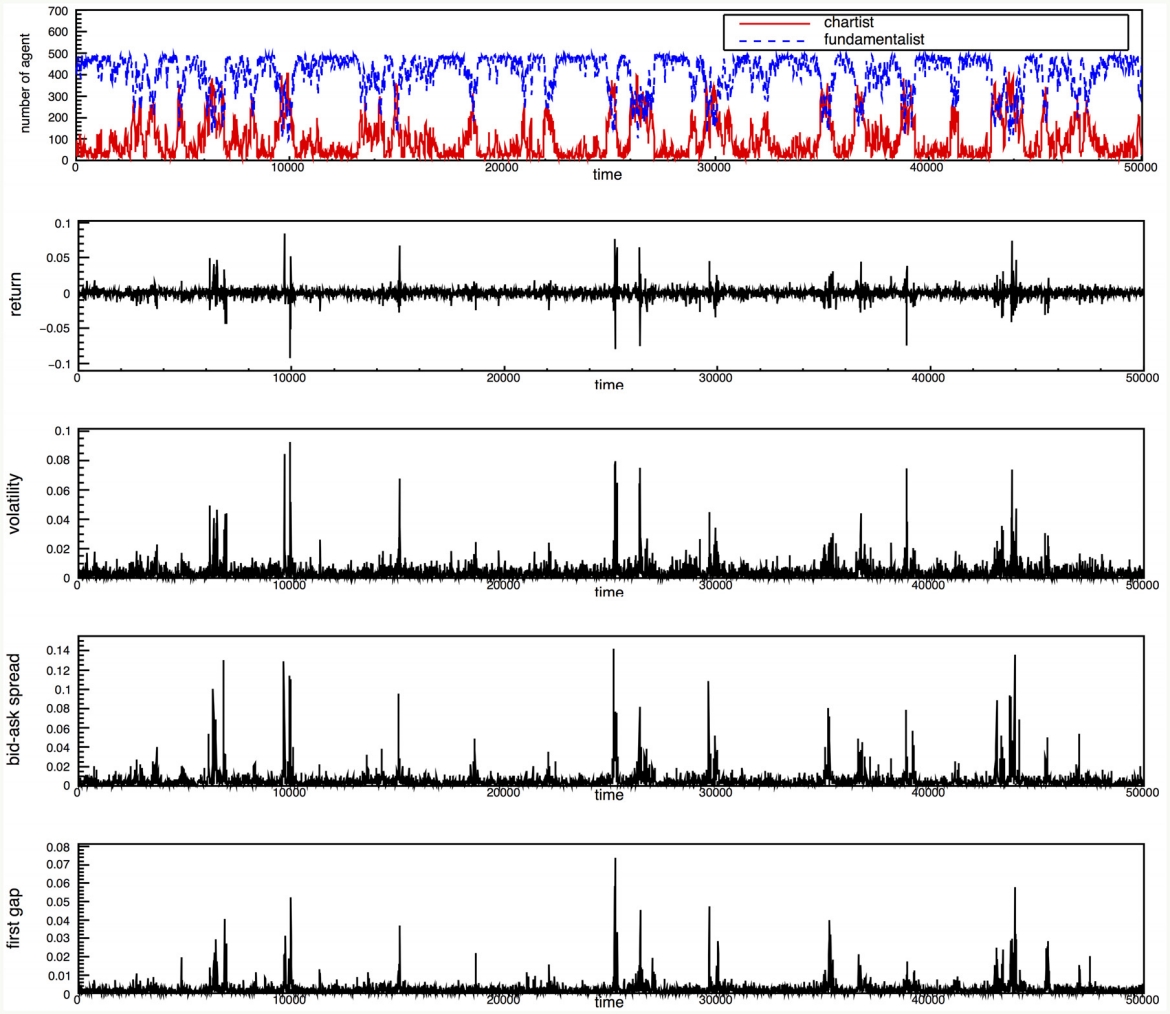
The dynamics of the agent population and the order book. From the top to the bottom, the figures show the dynamics of the type of agent in the market, returns, volatility, the bid-ask spread and the first gap as a function of time. In the top figure, the solid red line represents the number of chartists, and the dashed blue line represents the number of fundamentalists in the market.

Fig 2(a) depicts a large fluctuation over time, which indicates that an intermittent increase in the number of chartists tends to deviate from the efficient market, which consists only of fundamentalists. Deviation from this behavior, however, supports the efficient market hypothesis. Fig 2(b)–2(d) show the temporal movement for the return time series, volatility, the bid-ask spread, and the first gap, respectively. The bid-ask spread is calculated by the difference between the best ask and the best bid price, and the first gap corresponds to the difference between the best bid(ask) and the next best bid(ask). In Fig 2(b)–2(d), we find that the temporal movement of the four variables should be caused by the ratio of chartists in the market, which has also been found previously by both theoretical models and ABM [27, 28, 35]. Based on the results from Fig 2, the degree of information asymmetry induced from the chartist traders plays an important role in the transition behavior among diverse market states. In the Supporting Information, we also calculate the statistical and dynamic properties for the four variables using detrended fluctuation analysis and the probability density function; similar to the results from previous works [53–61], we find that the long-range correlation observed in all of the data sets, except for the return time series and the distribution function, follows a power-law distribution function regardless of data set. We also verify the usefulness of the proposed model by analyzing the time series properties of the artificial data sets generated by the ADAM.

**Fig 3:**
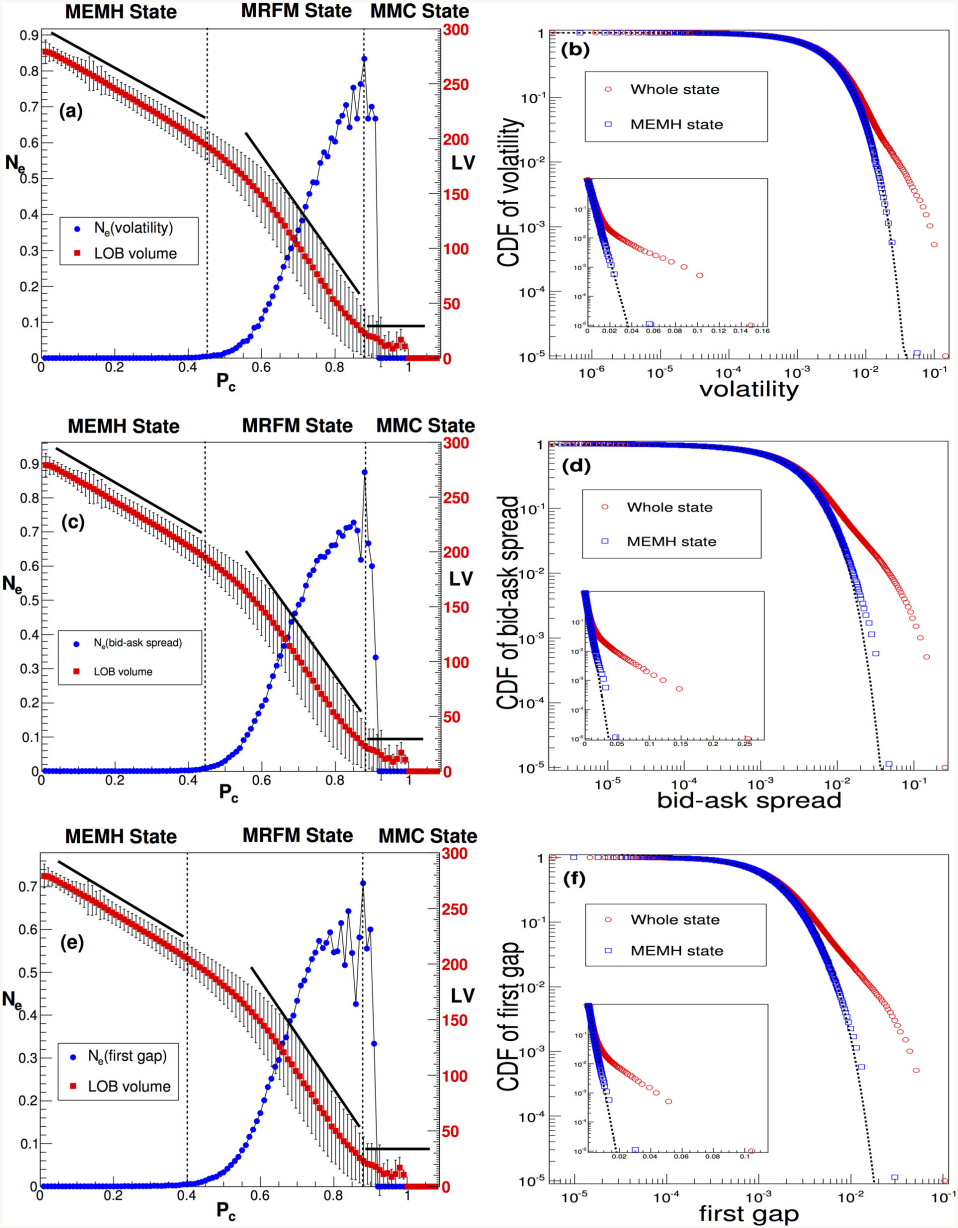
Market states according to *P*_*c*_—(a)(c)(e). There are *N*_*e*_, and LV(limit order book volume) as a function of *P*_*c*_. *N*_*e*_ denotes the number of extreme events normalized by the number of total events. We define a financial crisis event as a value larger than 4 *σ*. *σ* is the standard deviation of the given time series. A blue filled circle depicts *N*_*e*_, and a red filled square depicts the limit order book volume. According to *P*_*c*_, we classify the market into three distinct states: MEMH(Mimicking Efficient Market Hypothesis), MRFM(Mimicking Real Financial Market), or MMC(Mimicking Market Collapse). (b)(d)(f) There are CDFs(Cumulative Distribution Functions) of volatility, the bid-ask spread and the first gap in an MEMH state and the entire state. A red blank circle represents the CDF of an entire state. The blue-black square represents the CDF of an MEMH state. A dashed black line represents an exponential distribution function.

Given the significant role that heterogeneous investors in the financial market play in creating a complicated market structure in comparison with the EMH market with homogeneous traders, it is natural to expect that the degree of heterogeneity of traders is essential to generating diversity in financial market conditions. To investigate the validity of this hypothesis in terms of understanding the fundamental features of asset prices in diverse market states, we analyze whether the various market states, including normal, abnormal, and collapsed, are determined by the ratio of chartists as the source of abnormal market conditions. To analyze the relationship between the heterogeneity of traders and market states, we divide the market microstructure data of the ADAM model into a sub-data set based on the ratio of chartist traders, *P*_*c*_. As Fig 3(a), 3(c) and 3(e) show, for three quantities, volatility, bid-ask spread, and the first gap, the ratio of chartist traders, *P*_*c*_, is an important factor that describes diverse market states. In Fig 3(a), 3(c) and 3(e), we depict the probability of extreme events, *N*_*e*_, and the volume of limit order books, LOB, as a function of the *P*_*c*_ of those data sets. *N*_*e*_ is measured by the ratio of the events exceeded by four standard deviations, 4*σ*. In all cases, *N*_*e*_ and the volume of LOB is significantly related to *P*_*c*_, which indicates that in three quantities, *P*_*c*_ plays an important role in terms of determining the market state. In Fig 3(a), 3(c) and 3(e), we find that, regardless of data set, three distinct market states are observed that define each market state as mimicking the efficient market hypothesis(MEMH, $0\leq P_{c}^{\text{volatility}}\leq 0.45$, $0\leq P_{c}^{\text{bid-ask}\;\text{spread}}\leq 0.44$, and $0\leq P_{c}^{\text{first-gap}}\leq 0.40$), mimicking the real financial market(MRFM, $0.45<P_{c}^{\text{volatility}}<0.85$, $0.44<P_{c}^{\text{bid-ask}\;\text{spread}}<0.85$, and $0.40<P_{c}^{\text{first-gap}}<0.85$), and mimicking a market collapse(MMC, *P*_*c*_ ≥ 0.85). The MRFM states are defined by a threshold value larger than *N*_*e*_ = 0.005. These results show that the MEMH state is similar to the efficient market proposed by Fama [2]. In an MEMH state, fundamentalists are more prevalent than chartists in the market. With a substantial number of fundamentalists, the market price converges to a fundamental value and reflects full rationality. The volume of LOB increases because of the large number of limit orders submitted by fundamentalists in the MEMH state, indicating that fundamentalists can play a role as liquidity providers. An MEMH state in the specific range of *P*_*c*_ implies that the efficient market is only an ideal state or one type of state(out of a variety of possible states) in the financial market. In an MEMH state, the limit order volume decreases with increases of *P*_*c*_ (Fig 3(a), 3(c) and 3(e)), which implies that most market order submissions or transactions are performed by chartists. For an MEMH state in which the fundamentalists are more dominant than chartists in the market, the probability density function (PDF) of those variables follows a Gaussian distribution function, which is similar to the EMH proposed by Eugene F. Fama [2] and indicates that the market price converges to a fundamental value that should be generated by fully rational agents based on complete information. The essential features of market prices generated only by fundamentalists are similar to those of the EMH (see S1 File). For an MRFM state, there is a positive correlation between the *N*_*e*_ of three variables and the *P*_*c*_ value, and the PDF in Fig 3(b), 3(d) and 3(f) follows a power-law distribution that is observed in a real financial market, which reveals that an increasing number of chartists generates extreme events that are frequently found in the real financial markets. This finding indicates that the prices in an MRFM state have trends or fashions and deviate from efficient market states, as proposed by Robert J. Shiller [4]. In other words, because there are more chartists than fundamentalists in the market, the market price becomes largely separated from the fundamental value (random walk). We also find that the volume of the limit order book (LOB) and *P*_*c*_ shows a negative relationship in Fig 3(a), 3(c) and 3(e). In an MMC state, we find that *N*_*e*_ drops to zero due to the shortage of transactions, and the volume of LOB is almost zero. According to these results, the chartists should behave as liquidity takers and can sharply increase the liquidity risk. An MMC state created by chartists will ultimately lead to a market collapse because of the liquidity shortage.

**Fig 4:**
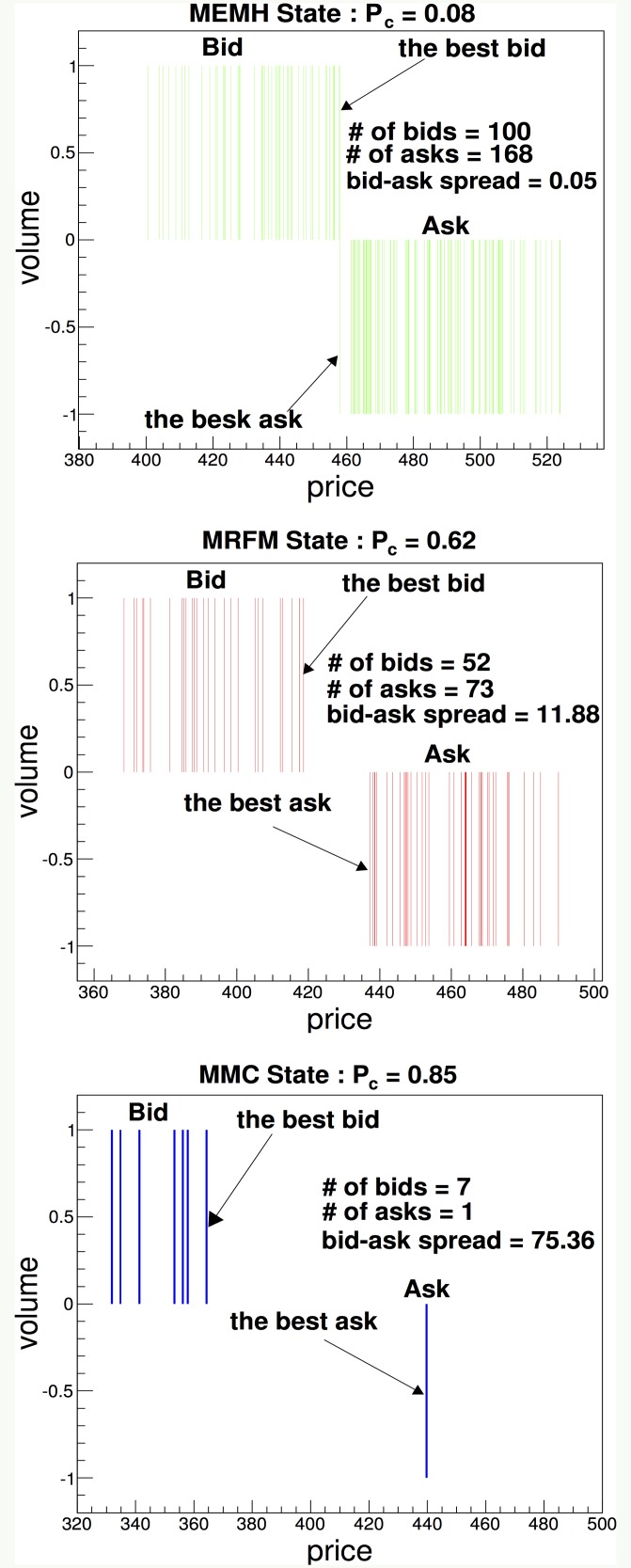
Snapshots of limit order books in three market states. The figure shows snapshots of the limit order books in the MEMH, MRFM and MMC states. A negative volume indicates the volume of asks. The bid-ask spread is defined by the difference between the best ask, which is the lowest ask among limit orders, and the best bid, which is the highest bid among limit orders.

To analyze the characteristics of the limit order book for the three distinct market states, we show snapshots of the LOB of each state. Fig 4(a), 4(b) and 4(c) show the LOB of the MEMH(*P*_*c*_ = 0.08), MRFM(*P*_*c*_ = 0.62) and MMC(*P*_*c*_ = 0.85) states, respectively. In Fig 4(a), 4(b) and 4(c), we find that the bid-ask spread and the width between limit orders increase as *P*_*c*_ increases; thus, *P*_*c*_ is the smallest in the MEMH state and has the largest value in the MMC state, which is characterized by a liquidity shortage or liquidity evaporation. In Fig 4(b), we find that a suitable ratio of chartists increases the liquidity of transactions among agents and that it will reduce the liquidity risk. Thus, the stability of the financial market should be controlled by the ratio of chartists in the entire population of traders. Previous empirical studies have reported that the bid-ask spread in a financial crisis is larger than it is before the crisis [50, 51]. It has been reported that specialists in the NYSE (New York Stock Exchange) had trouble executing trades because of a dramatic shortage of limit orders on Black Monday, October 19,1987 [52]. Our model is consistent with these empirical findings, including the liquidity shortage on Black Monday.

**Fig 5:**
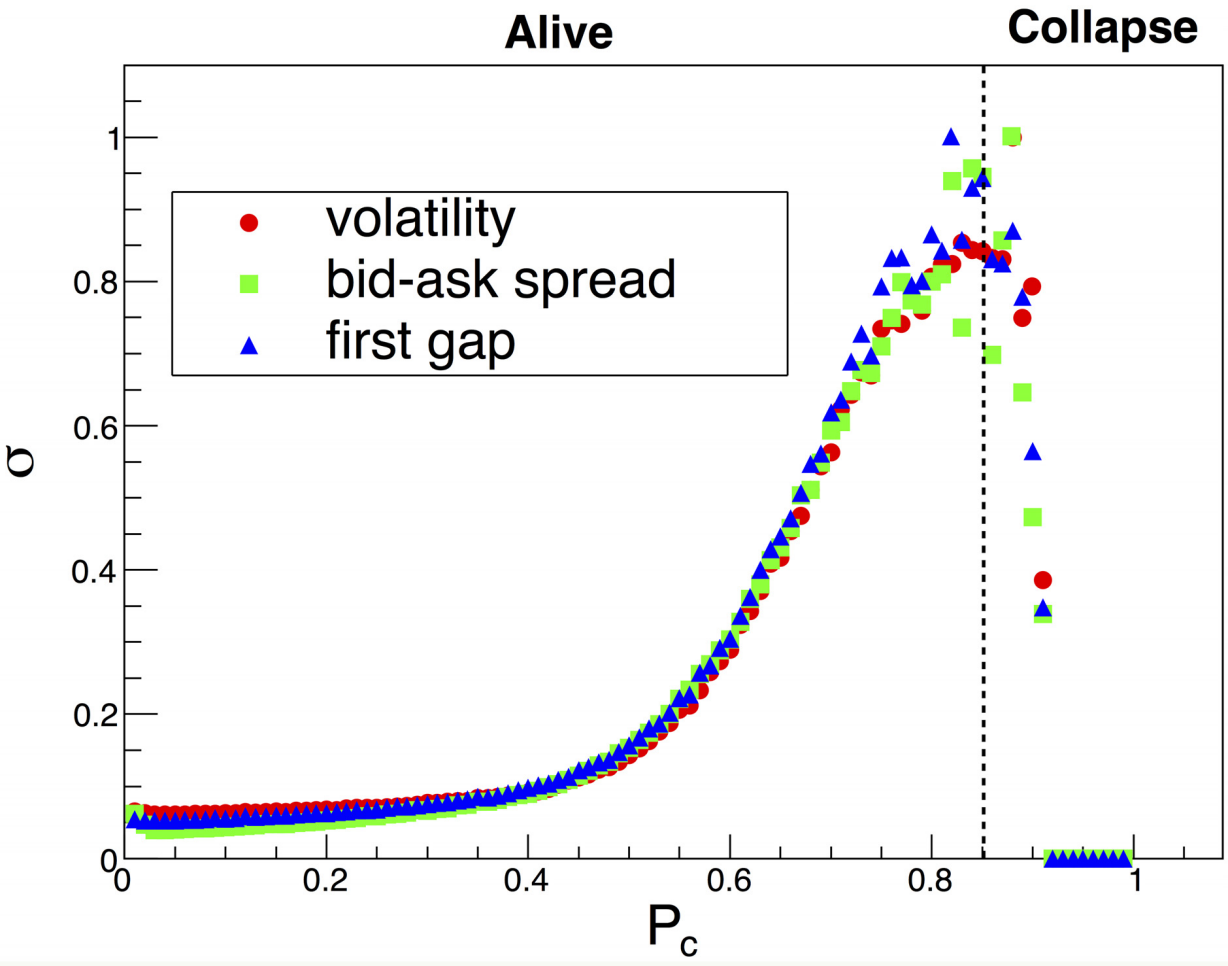
Phase transition behavior according to *P*_*c*_—*σ* (Standard deviation) of volatility, the bid-ask spread and the first gap as a function of *P*_*c*_. The maximum value of *σ* is normalized by 1.

The standard deviation of volatility, the bid-ask spread and the first gap as a function of *P*_*c*_ increase and have peak points at approximately 0.85 (Fig 5). All of the maximum standard deviation values of these quantities are normalized by 1. When *P*_*c*_ is greater than 0.85, the standard deviation of the three quantities dramatically decreases and is close to zero in Fig 5. Our results suggest that the behavior of LOB appears to be a transition from an alive state to a collapsed state at approximately *P*_*c*_ = 0.85, which is similar to the phase transition behavior described by previous research [62].

We observe various stylized facts in asset returns and market microstructures such as power-law tails, a long memory of volatility, and first gap and aggregational Gaussianity [53–61]. To confirm the effect of the chartists, we perform an analysis of a homogeneous equilibrium market that consists of only fundamentalists. The results show that market prices converge to the fundamental value, and the market state does not deviate from the MEMH state during the simulation. MRFM, MMC and stylized facts are not observed in the homogeneous equilibrium market. The details of the analysis of the stylized facts and the results of the analysis of a homogeneous equilibrium market can be found in the Supporting Information (S1 File).

## Discussion and Conclusion

Understanding asset pricing is a crucial issue in financial markets; however, because there is no adequate theory to explain it that includes all aspects of the price mechanisms, most of the theories reflect only the partial features of the manner in which asset prices are determined by the investment strategies of agents.

Indeed, when applying important elements, including the heterogeneity of traders and the double auction market as the trading system, to an artificial market, one must also consider the diversity of market states. In this study, we have suggested the ADAM model to illustrate the diversity of the financial markets from an efficient market to behavioral finance. The ADAM model consists of heterogeneous traders, including fundamentalists and chartists, and provides a differentiated framework compared with previous ABMs. Our key finding is that the diversity of the financial market, including the MEMH, MRFM, and MMC, is determined primarily by the ratio of chartists, *P*_*c*_, among all of the traders in the market.

The ADAM framework developed herein offers explanations and raises questions that can deepen our understanding of asset pricing models in financial markets. For example, although the ADAM cannot be considered a multi-asset pricing model, the framework we develop can explain the diversity of financial market states and can provide a framework with which to address the role of the heterogeneity of agents.

In summary, our findings argue that many aspects of market states can be explored numerically using the ABM if we combine the heterogeneity of traders and the double auction market as trading systems, thus creating the potential for a more in-depth understanding of financial markets.

## Supporting Information

S1 File More details of transition probabilities and additional result of analysis related to finding of some *stylized facts* in financial market using ADAM. (PDF)
