# Supplementary material for: Understanding Financial Market States Using an Artificial Double Auction Market
Source: PLoS One. 2016 Mar 31;11(3):e0152608. doi: 10.1371/journal.pone.0152608 (PMC4816384; doi:10.1371/journal.pone.0152608)
Supplement: S1 File — (PDF) [file pone.0152608.s001.pdf]

Understanding Financial Market States Using Artificial Double Auction Market

Kyubin Yim[1,◑], Gabjin Oh[2,◑*], Seunghwan Kim[1,◑],

1 Nonlinear and Complex System Laboratory,Department of Physics, Pohang University of Science and Technology, Pohang 790-784, Republic Of Korea
2 Division of business administration, Chosun Univesity, Gwangju 501-759, Republic Of Korea

◑These authors contributed equally to this work.
*** phecogjoh@gmail.com**

Supporting Information

The switching rules of agents' opinions

In this paper, we apply the modified transition rules of agents' opinions which are based on Lux and Marchesi(1999) [1]. The transition probabilities introduced in Lux and Marchesi(1999) [1] denoted by $\pi_{A,B}\Delta t$. $\pi_{A,B}$ is the transition rate from type B to type A.

$$\pi_{+-} = v_1 \frac{n_c}{N} \exp(U_1), \pi_{-+} = v_1 \frac{n_c}{N} \exp(-U_1),$$

$$U_1 = \alpha_1 x + \frac{\alpha_2}{v_1} \frac{dp/dt}{p}$$

$$\pi_{+f} = v_2 \frac{n_+}{N} \exp(U_{2,1}), \pi_{f+} = v_2 \frac{n_f}{N} \exp(-U_{2,1})$$

$$\pi_{-f} = v_2 \frac{n_-}{N} \exp(U_{2,2}), \pi_{f-} = v_2 \frac{n_f}{N} \exp(-U_{2,2})$$

$$U_{2,1} = \alpha_3 \{ \underbrace{\frac{r + (1/v_2)(dp/dt)}{p} - R}_{\text{profit of optimistic}} - \underbrace{s|\frac{p_f - p}{p}|}_{\text{fundamentalists' profit}} \}$$

$$U_{2,2} = \alpha_3 \{ \underbrace{R - \frac{r + (1/v_2)(dp/dt)}{p}}_{\text{profit of pessimistic}} - \underbrace{s|\frac{p_f - p}{p}|}_{\text{fundamentalists' profit}} \}$$

where $x = (n_+ - n_-)/n_c$,N, n_c, n_+, n_-, n_f denote the number of agents, chartists, optimistic agents, pessimistic agents, and fundamentalists.p denotes the current market price, and p_f is the current fundamental value. The subscript $+, -, f$ denotes the agent type, e.g., optimist, pessimist, or fundamentalist. The mechanism that can change the agent types in transition probability consists of a herding and profit strategy. If the fraction of optimistic agents (n_+/N) which is related to herding strategy increases in the market, the transition probabilities from other types to optimistic agents(π_{+-}, π_{+f}) increase. In addition, if the profit of optimistic agents

increases, the transition probabilities from other types to optimistic agents increases. The other types such as pessimists and fundamentalists also have similar behaviors.

To avoid absorbing state($n_c = 0$ or $n_f = 0$) in simulation, we apply a rule that an agent with an opinion in a population less than 0.8% in the total population can not change her own opinion into other opinions.

We assume that the investment time horizon of each agent type is different. Chartists have shorter investment time horizon than fundamentalists. However, in the transition probabilities in Lux and Marchesi(1999) [1], the heterogeneous investment time horizon of an agent is not considered. In other words, dp/dt is calculated during homogeneous investment time horizons. However, in the real financial market, investors have heterogeneous information sets. Each investor cannot help but have a different strategies due to the differences between information sets that agents have. With the notion of heterogeneous information sets between agents, we modify this term dp/dt, which can reflect the investment time horizon of heterogeneous agent types. dp/dt is calculated by the average value of $\Delta p/\Delta t$ during agents' own investment time horizon $[t - \tau^i$,t).

The values of the parameters are set as follows: N (number of agents) = 500, $v_1 = 2$, $v_2 = 0.6$, $\alpha_1 = 0.6$, $\alpha_2 = 1.5$, $\alpha_3 = 1$, $r = Rp_f (R = 0.0004)$, $s = 0.75$, $\Delta t = 0.01$ [time] ,$\tau^i = 3$ [time] investment time horizon for fundamentalists, $\tau^i = 1$ [time] for chartists.

For more details about other parameters in transition probabilities, see Lux and Marchesi(1999) [1].

Stylized facts in Artificial Double Auction Market

We analyze ADAM-generated data and observe various 'stylized facts' that were observed in previous empirical studies [2–11]. Fat-tails of market microstructure quantities such as absolute return, bid-ask spread and first gap, which were widely observed in previous empirical studies, are observed in the ADAM(Fig. 1). We fit tails of the CDF (Cumulative Distribution Function) of market microstructure quantities using a power-law function, $y \sim x^{-\alpha}$ and α is estimated by maximum likelihood estimation [12]. The fitted results are summarized in Table 1. Additionally, the distribution of return as time lag increases converges to the return distribution of fundamental value, which follows a normal distribution(Fig. 2(a)), which is called 'Aggregational Gaussianity', which is one type of 'stylized fact' in the financial markets [11].

To investigate the temporal correlation property of market microstructure, we use the DFA (Detrended Fluctuation Analysis) method introduced in Hu et al (2001) [13]. The steps of the DFA are as follows:

(i) We consider time series $x(i)$ $(i = 1, ..., N_{max})$ noise induced. We integrate the time series $x(i)$, and which is divided into boxes of equal size n.

$$y(j) = \sum_{i=1}^{j} [x(i) - < x >] \tag{1}$$

where

$$< x > = \frac{1}{N_{max}} \sum_{j=1}^{N_{max}} x_i \tag{2}$$

(ii) In each box, the integrated time series $y(i)$ is fitted by a polynomial function, $y_{fit}(i)$, which is called a local trend. For order-l DFA(DFA-1 if l=1, DFA-2 if l=2,

etc.), we could apply the l-order polynomial function for the fitting. Time series $y(i)$ is detrended by subtracting the local trend $y_{fit}(i)$ in each box, and we calculate the detrended fluctuation $Y(i)$.

$$Y(i) = y(i) - y_{fit}(i) \tag{3}$$

For a given box size n, the root mean square(rms) fluctuation function $F(n)$ is calculated as

$$F(n) = \sqrt{\frac{1}{N}\sum_{i=1}^{N_{max}} [Y(i)]^2} \tag{4}$$

(iii) We repeat the above computation for box sizes n to find a relationship between $F(n)$ and n, $F(n) \sim n^H$.

This power-law relationship indicates the presence of scaling between $F(n)$ and the box size n. The parameter H is called the Hurst exponent or the scaling exponent and represents the correlation property of the signal: If $H = 0.5$,the signal has no correlation (white noise); if $H < 0.5$, the signal has a mean-reverting property or a short memory; if $H > 0.5$, the signal has a persistent property or a long memory. In this paper, DFA-1 is used.

As a result, long memory is observed in volatility, the bid-ask spread, the volume and the first gap in the ADAM. In the return case, no memory is observed. These memory properties of the ADAM support previous previous empirical works [3, 5, 9, 14]. Fig. 3(a),(b) depicts the rms fluctuation functions $F(n)$ of these quantities. The results of the ADAM and previous empirical results are summarized in Table 2.

The case of the homogeneous equilibrium market

To distinguish the effect of chartists in the market, we investigate the homogeneous equilibrium market, which consists only of fundamentalists (i.e. there are no chartists in the market.) As Fig. 4shows, there is no volatility clustering or a significant peak of the bid-ask spread or the first gap. These results imply that there is only an MEMH state in the homogeneous equilibrium market. There are no fat-tails in the CDF of absolute return, the bid-ask spread and the first gap (Fig. 2 (b), Fig. 5). We measure the Hurst exponent using DFA-1. Fig. 3 (c) and (d) depict the rms fluctuation $F(n)$ of the given market microstructure time series. The Hurst exponents of these quantities are similar to 0.5 (Table 3), which indicates that there is no memory property in the homogeneous equilibrium market.

In short, when all agents are fundamentalists, abnormal market behavior, such as financial crisis does not occur. In additional, ‘stylized facts’ in the real financial market, such as long memory, fat-tails and Aggregational Gaussianity are not observed.

These results imply that chartists are the source of abnormal behavior in the ADAM.

Acknowledgments

This work was supported by the National Research Foundation of Korea(NRF) grant funded by the Korea government(MEST) (No.2013017095) and by the National Research Foundation of Korea Grant funded by the Korean Government (NRF- 4Q6 2014S1A3A2044643).

References

1. Lux T, Marchesi M. Scaling and criticality in a stochastic multi-agent model of a financial market. Nature. 1999;397:498–500.
2. Plerou V, Gopikrishnan P, Stanley HE. Quantifying fluctuations in market liquidity:Analysis of the bid-ask spread. Phy Rev E. 2005;71:046131.
3. Plerou V, Gopikrishnan P, Amaral L, Gabaix X, Stanley HE. Economic fluctuations and anomalous diffusion. Phys Rev E. 2000;62:3023–3026.
4. Farmer JD, Gillemot L, Lillo F, Mike S, Sen A. What really causes large price changes? Quantitative Finance. 2004;4:383–397.
5. Lillo F, Farmer JD. The key role of liquidity fluctuations in determining large price changes. Fluctuation and Noise Letters. 2005;5(2):209–216.
6. Weber P, Rosenow B. Order book approach to price impact. Quantitative Finance. 2005;5:357–364.
7. Gillemot L, Farmer JD, Lillo F. There?s more to volatility than volume. Quantitative Finance. 2006;6:371–384.
8. Ponzi A, Lillo F, Mantegna RN. Market reaction to a bid-ask spread change: A power-law relaxation dynamics. Phys Rev E. 2009;80:016112.
9. Liu Y, Gopikrishnan P, Cizeau P, Meyer Y, Peng CK, Stanley HE. Statistical properties of the volatility of price fluctuations. Phys Rev E. 1999;60:1390–1400.
10. Li W, Wang F, Havlin S, Stanley HE. Financial factor influence on scaling and memory of trading volume in stock market. Phys Rev E. 2011;84:046112.
11. Antypas A, Koundouri P, Kourogenis N. Aggregational Gaussianity and barely infinite variance in financial returns. Journal of Empirical Finance. 2013;20:102–108.
12. Clauset A, Shalizi CR, Newman MEJ. Power-law distributions in empirical data. SIAM Review. 2009;51:661–703.
13. Hu K, Ivanov PC, Chen Z, Carpena P, Stanely HE. Effects of trends on detrended fluctuation analysis. Phys Rev E. 2001;64:011114.
14. Oh G, Kim S, Um C. Statistical Properties of the Returns of Stock Prices of International Markets. Journal of Korea Physical Society. 2006;48:197–201.

Table 1. The fitted result of the power-law function-The fitted results of the power-law function, $y \sim x^{-\alpha}$. α is the power-law exponent. The elements of column ADAM(empirical result) show the results of the ADAM(empirical works).

	ADAM	Empirical Result
return(positive tails)	1.89 ± 0.03	2.19 [5]
return(negative tails)	2.03 ± 0.04	2.17 [5]
bid-ask spread	1.53 ± 0.01	3.0 [3]
first gap	1.78 ± 0.02	1.5 - 3.0 [4]

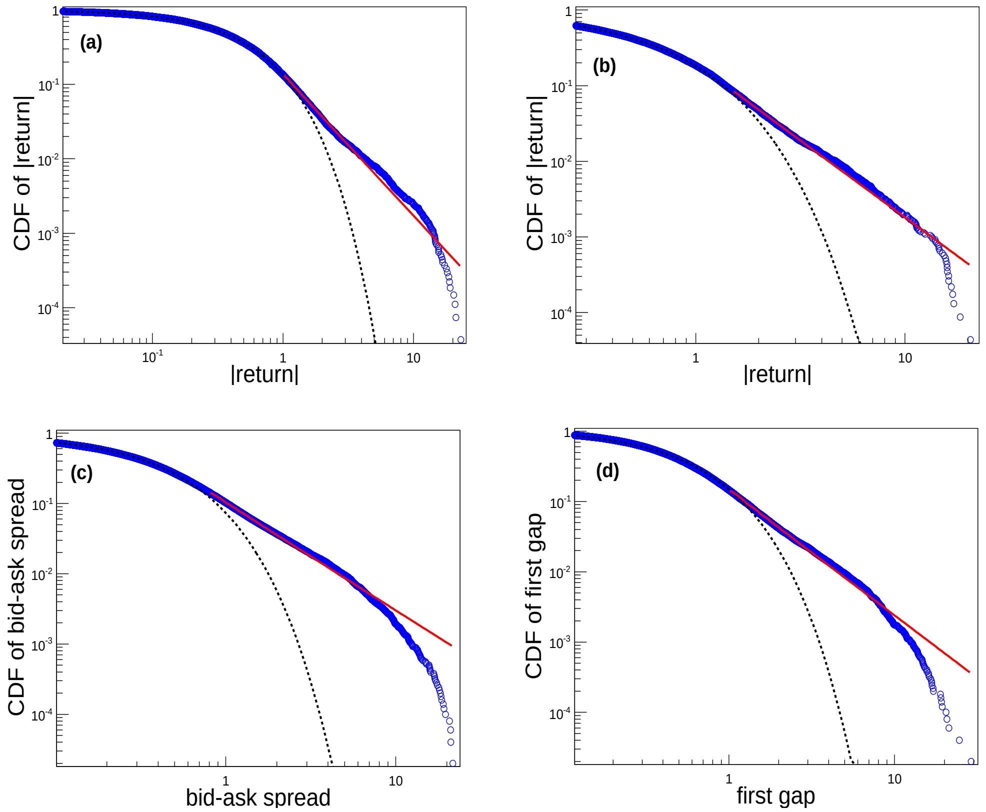

Figure 1. CDF of market microstructure variables-(a)(b)(c)(d)CDFs of positive return, negative return, bid-ask spread and first gap. The dashed black line represents an exponential distribution function. The solid red line represents the fitted power-law function using $y \sim x^{-\alpha}$.The values of the fitted α are summarized in Table 1

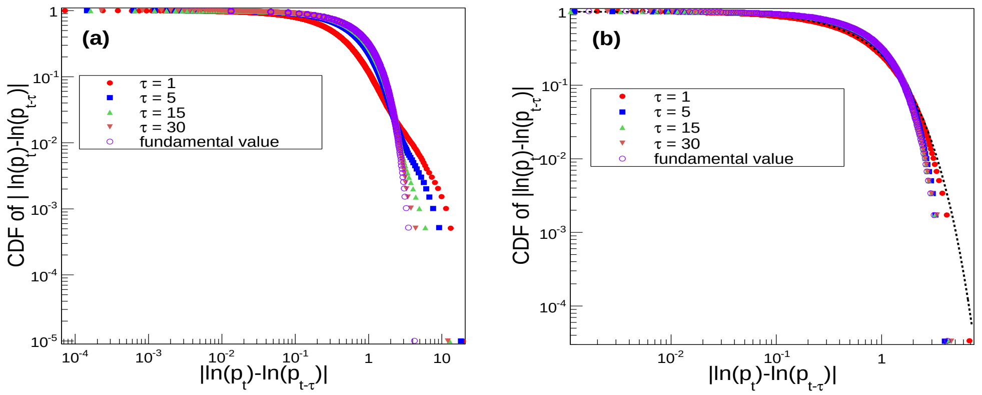

Figure 2. Aggregational Gaussianity-(a) CDF of |return|. Return is defined by r(t) = $\ln p(t) - \ln p(t - \tau)$. $p(t)$ denotes the market price at time t. (b) the CDF of |return| in a homogeneous equilibrium market(i.e., all agents are fundamentalist in the market). The dashed black line represents an exponential distribution function.

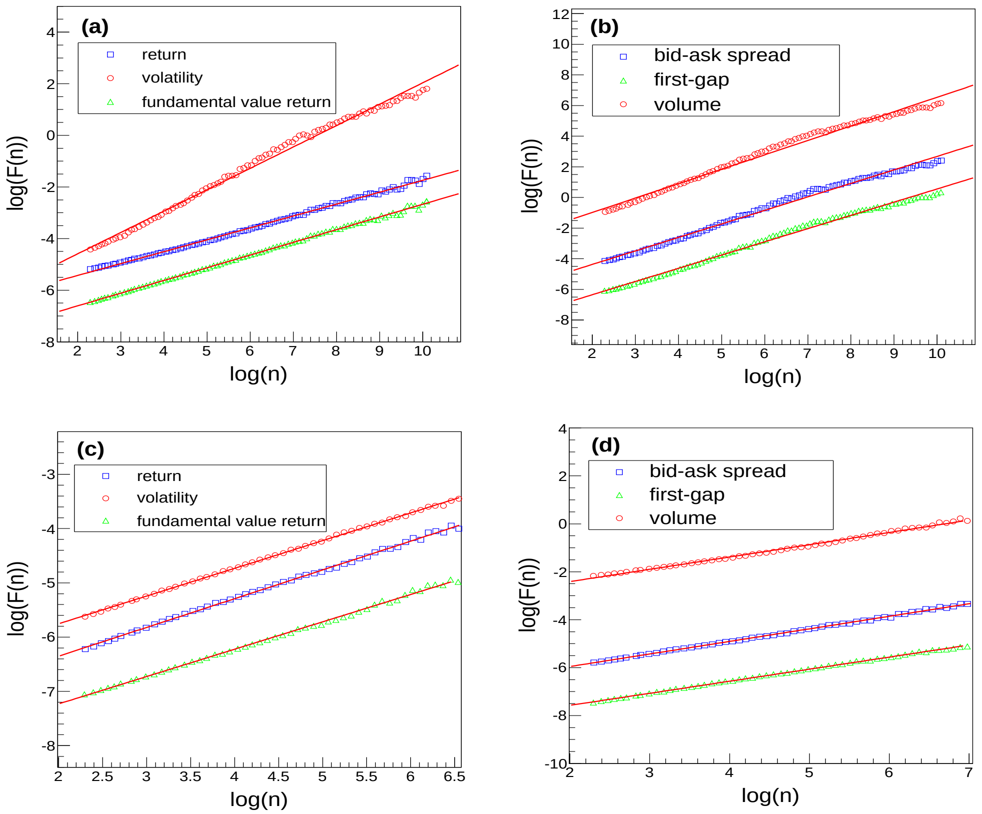

Figure 3. The rms fluctuation function - (a)(b) F(n) denotes the rms fluctuation function. The slope of the rms fluctuation function indicates the Hurst exponent. The Hurst exponents measured are summarized in Table 2.(c)(d). F(n) is the rms fluctuation function in the homogeneous equilibrium market. The Hurst exponents measured in this case are summarized in Table 3.

Table 2. The Hurst exponent results of the ADAM and the comparison to empirical works - The elements of column ADAM(empirical result) show the results of the ADAM(empirical works). The words in parenthesis at Memory Type indicate the memory type observed in empirical works.[FV : fundamental value]

	ADAM	Empirical Result	Memory Type
return	0.46 ± 0.01	0.5 [14]	No(No)
volatility	0.82 ± 0.01	0.67 [9]	Long(Long)
bid-ask spread	0.87 ± 0.01	0.73 ± 0.01 [3]	Long(Long)
first gap	0.87 ± 0.01	0.76 [5]	Long(Long)
FV return	0.49 ± 0.01		No
volume	0.94 ± 0.01	0.64 - 0.7 [10]	Long(Long)

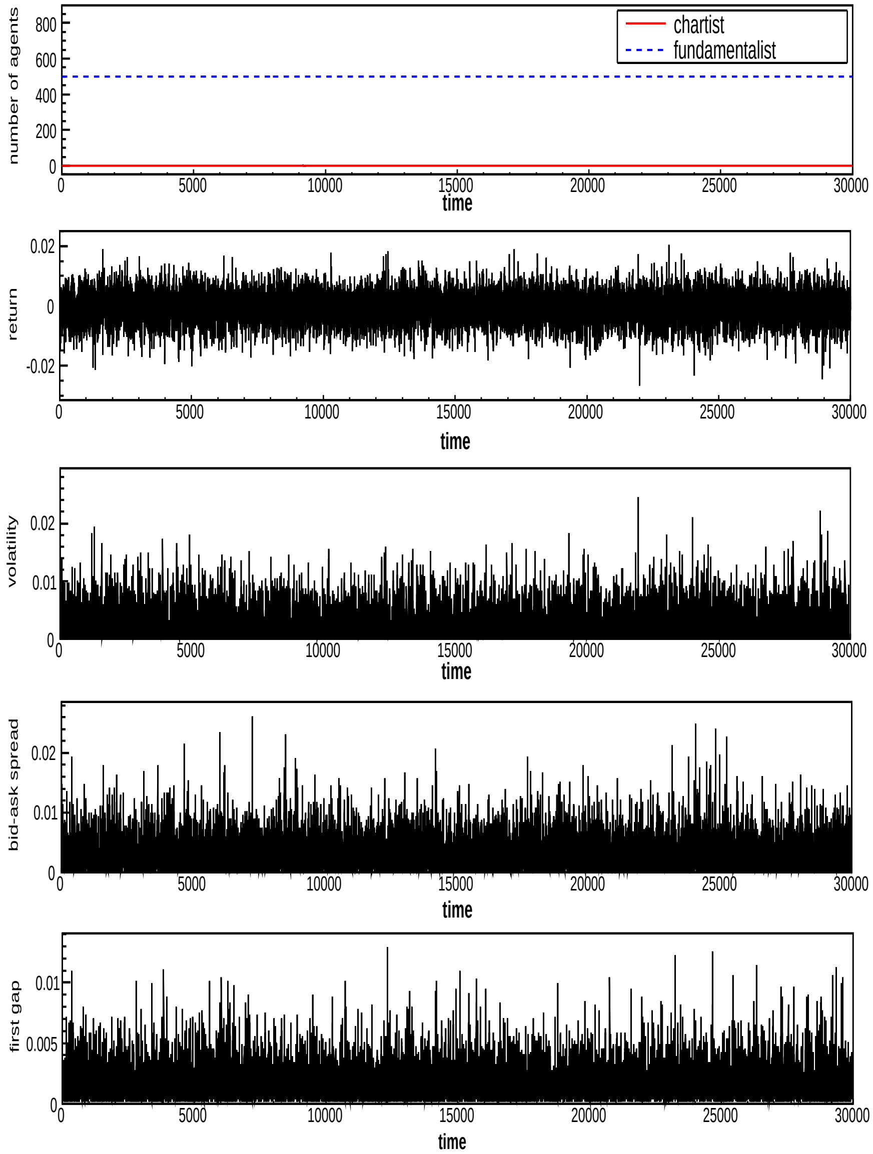

Figure 4. The dynamics of agents population and order book in the homogeneous equilibrium market - From the top to bottom in figures, the population dynamics of agents' types in the market, return, volatility, bid-ask spread and first gap as a function of time in the homogeneous equilibrium market. In the top figure, the solid red line shows the number of chartists, and the blue dashed line shows the number of fundamentalists in the market.

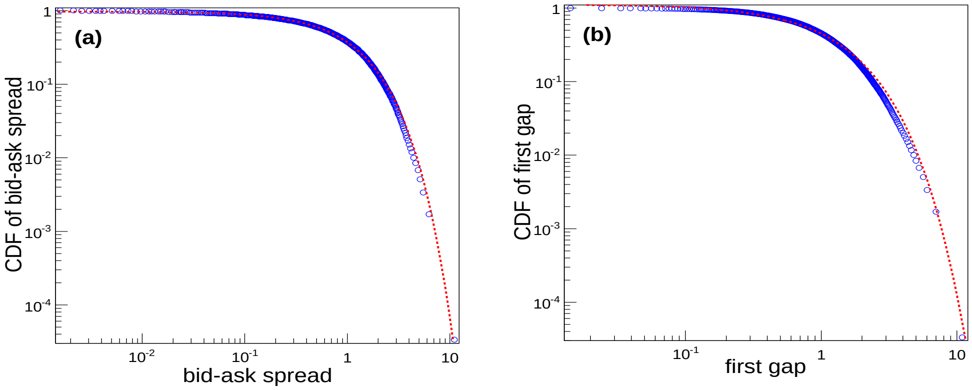

Figure 5. The CDF of the bid-ask spread and the first gap in the homogeneous equilibrium market - (a) CDF of bid-ask spread. (b) CDF of first gap. The dashed red line shows an exponential distribution function.

Table 3. The Hurst exponents in the homogeneous equilibrium market of the ADAM - There is no memory in market microstructures in this case.[FV : fundamental value]

	ADAM	Memory Type
return	0.52 ± 0.01	No
volatility	0.51 ± 0.01	No
bid-ask spread	0.52 ± 0.01	No
first gap	0.50 ± 0.01	No
FV return	0.50 ± 0.01	No
volume	0.51 ± 0.01	No
